# Nationwide outbreak of *Salmonella* Enteritidis linked to consumption of pumpkin seeds, France, 2026

**DOI:** 10.2807/1560-7917.ES.2026.31.38.2600796

**Published:** 2026-09-24

**Authors:** Gabrielle Jones, Edith Laurent, Erica Fougère, Nathalie Fredricksen, Caroline Kojfer-Lomont, Lorraine Puzin, Sophie Belichon, Anastasia Petrova, Athénaïs Bur, Henriette de Valk, Leonor Silveira, Christian Kornschober, Catherine Ragimbeau, Laetitia Bonifait, Alexandra Moura

**Affiliations:** 1 Santé publique France, Infectious Diseases Division, Saint-Maurice, France; 2 Santé publique France, Regional Division – Auvergne-Rhône-Alpes, Lyon, France; 3 French General Directorate for Food, Paris, France; 4 National Reference Laboratory of Gastrointestinal Infections, National Institute of Health Doutor Ricardo Jorge, Lisbon, Portugal; 5 National Reference Centre for Salmonella Austria, Institute for Medical Microbiology & Hygiene Graz, Austrian Agency for Health and Food Safety, Graz, Austria; 6 Laboratoire National de Santé, Microbiology department, Dudelange, Luxembourg; 7 French Agency for Food, Environmental and Occupational Health & Safety, Ploufragan-Plouzané-Niort Laboratory, National Reference Laboratory for *Salmonella*, Ploufragan, France; 8 Institut Pasteur, Université Paris Cité, Enteric Bacterial Pathogens Unit, National Reference Centre for *Escherichia coli*, *Shigella* and *Salmonella* & WHO Collaborating Center for *Salmonella*, Paris, France

**Keywords:** *Salmonella* Enteritidis, pumpkin seeds, foodborne outbreak, France

## Abstract

A nationwide outbreak of *Salmonella* Enteritidis is ongoing in France with 81 patients from 3 May to 12 August 2026. Median age was 35 years, and 56 case-patients were female. Investigations identified pumpkin seeds grown in France as the source. A food isolate reported on EpiPulse reinforced initial hypotheses. Recall-withdrawal was initiated on 28 August. Traceback continues including for export. Pumpkin seeds represent a novel *Salmonella* risk that should be considered in seed production and by public health authorities.

On 30 July 2026, the National Reference Centre for *E. coli*, *Shigella* and *Salmonella* (NRC-ESS) at Institut Pasteur and Santé publique France identified an increase in *Salmonella* isolates belonging to a genomic cluster of *Salmonella enterica* subsp. *enterica* serovar Enteritidis (SE) cgMLST type HC5_2041/HC2_710439 (EnteroBase cgMLST scheme) comprising 49 isolates since May 2026. Case characteristics were unusual in that cases were predominately female (32 cases), had a median age of 32 years and were disproportionately from a single region (17/49 cases). We here describe the microbiological, epidemiological and traceback investigations conducted to identify the outbreak source and implement appropriate control measures.

## Microbiological, epidemiological and traceback investigations

Microbiological investigations were conducted at the NRC-ESS, which receives *Salmonella* isolates from private and hospital laboratories. Isolates undergo routine whole genome short-read sequencing. Sequence analyses and cluster affiliation are performed using the core genome multilocus sequence typing (cgMLST) scheme of 3,002 loci and the hierarchical clustering implemented in EnteroBase [1]. The delay from strain isolation at the primary laboratory and sequencing results is around 4 weeks.

In order to generate hypotheses on a possible common source of infection, Santé publique France conducted epidemiological investigations by telephone with cases (or case guardians if the cases were minors), using a trawling *Salmonella* questionnaire of 25 pages enquiring about food and environmental exposures. A confirmed case was defined as a person with an SE isolate of cgMLST type HC5_2041/HC2_710439.

The French General Directorate for Food (DGAl) conducted traceback investigations from reported food exposures and retail outlets identified from the questionnaires, as well as data from supermarket loyalty cards when available. Once a suspected food item was identified, traceback was conducted from supplier data, as well as on-site inspections by the local Departmental division attached to the DGAl (Lot and Garonne department; DDETSPP47).

Food microbiological analyses were conducted by specialised departmental laboratories for *Salmonella* detection and isolation, or by the National reference laboratory (NRL) for *Salmonella* (Anses-Ploufragan). Sequencing of food isolates was conducted by the NRL.

## Hypothesis generation

Hypothesis generating interviews quickly identified a majority of cases reporting food shopping at a specific supermarket chain (Supermarket A; 7/9 cases initially interviewed). However, no specific food item was identified, and foods typically associated with *Salmonella* outbreaks (raw milk cheeses, cold cuts, ground beef) were uncommon. Poultry and eggs were more often consumed, but with no common place of purchase.

As investigations continued, an unusually large proportion of cases reported consuming different nuts and seeds (8/14 cases), almost all (6/8 cases) from supermarket A. The trawling *Salmonella* questionnaire includes some specific nuts and seeds, but no single item was predominant among cases. However, this proportion of consumers appeared unusual compared with previous outbreak investigations.

We collected loyalty card numbers with case consent. While loyalty card data are typically requested for the month before symptom onset, as nuts and seeds can be conserved for long periods, the DGAl requested data from March 2026 forward.

**Figure 1:**
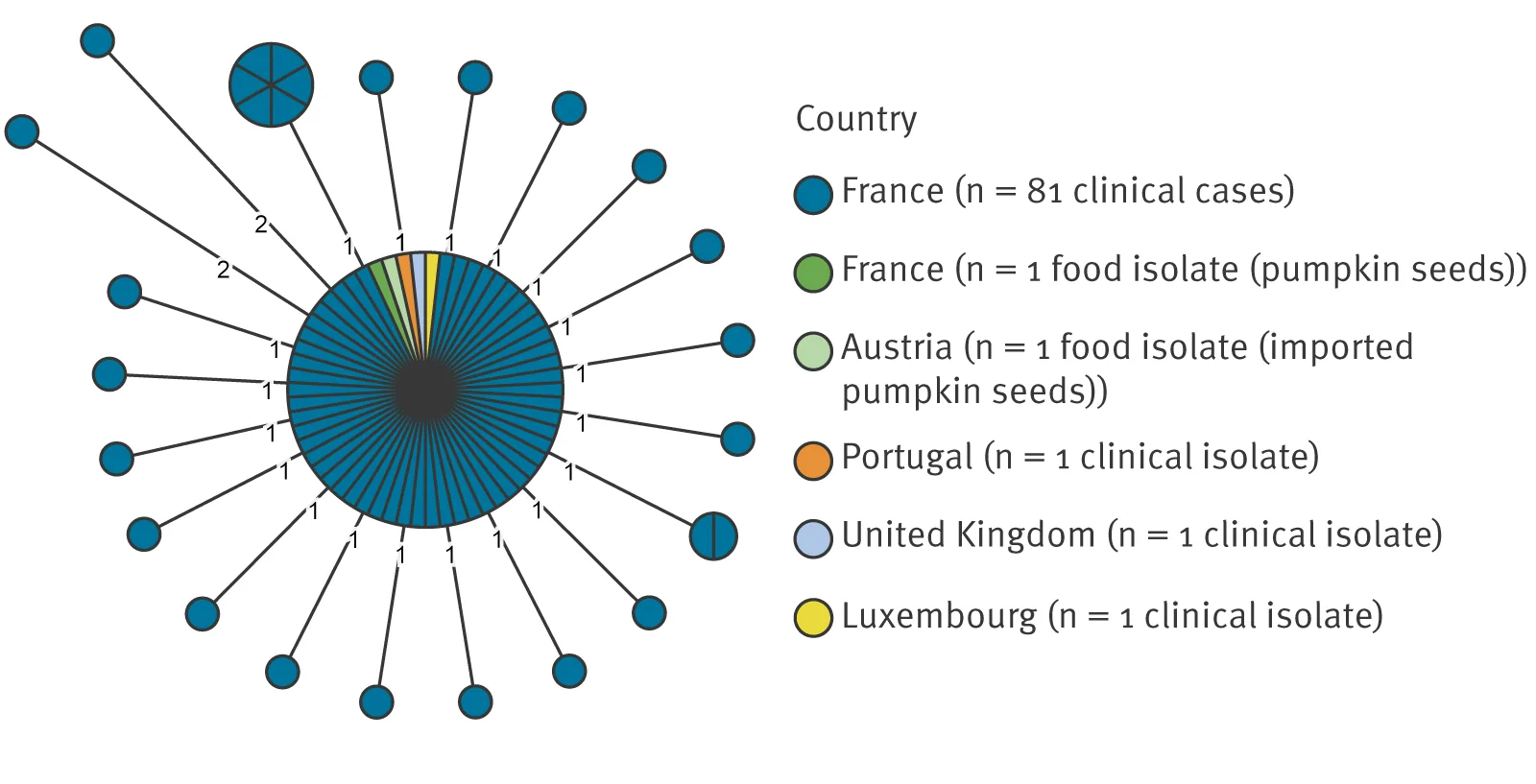
Minimum spanning tree based on the cgMLST profiles (Enterobase scheme of 3,002 loci) profiles of *Salmonella* Enteritidis HC5_2041/HC2_710439 isolates within the outbreak cluster, France, May–August 2026, (n = 86)

We posted on 18 August a message on EpiPulse, an online platform for European health authorities to exchange information on outbreaks, and received a response from Austria on the same day reporting a food isolate obtained in June 2026 from pumpkin seeds imported from Poland (identical cgMLST profile as the reference French isolate) (Figure 1). Loyalty card data from Supermarket A for five cases became available on 19 August. Numerous purchases of various nuts and seeds were identified on all cards, but pumpkin seeds were common to all five cases.

cgMLST: core genome multilocus sequence typing.

All genomes were shared through Enterobase. The nodes represent each cgMLST profile and are proportional to the number of isolates. Colour represents the country of isolation. The numbers on the branches indicate the number of cgMLST allelic differences between profiles. Updated 9 September 2026. For non-French isolates, the sequencing data was sent by each country for analysis by the NRC-ESS.

From 20 August, we asked all new cases to answer questions on a detailed list of nuts and seeds, including pumpkin seeds, and previously interviewed cases were called back. We confirmed consumption of pumpkin seeds alone or in nut/seed mixes for 12 of 16 cases, purchased pre-packaged at Supermarket A for 10 of 12 cases.

## Food traceback and control measures

Traceback investigations identified a single producer of pumpkin seeds, Producer X, who supplied Supermarket A. Producer X supplied the pumpkin seeds in large bags labelled with a lot number to two French repackaging contractors, who repackaged them into smaller containers for sale in bulk, or pre-packaged them ready-to-buy (alone or in mixes) for Supermarket A. For seeds pre-packaged by a contractor, the lot number corresponded to the product’s best-by date. The data from the five available loyalty cards identified numerous possible batches from the documented purchases.

Food samples of pumpkin seeds from supermarket A in the original packaging were taken at the homes of three cases. All three samples tested positive for SE (on 26 August, 2 and 11 September), and sequencing confirmed clustering with cases. A wider search of the NRL database did not identify any historical isolates of SE isolated from nuts and seeds.

On 28 August, the supplier of Supermarket A initiated recall and withdrawals of all pumpkin seed batches on the market since 1 March, accompanied by a press release [2-4].

Traceback investigations identified that the incriminated pumpkin seeds were exclusively grown from seedlings imported from Austria, cultivated and processed in France. Currently, traceback has not been able to identify a direct or indirect link between Producer X and pumpkin seeds from Poland. On-site inspections identified two surface samples from the processing plant were positive for SE (sequencing underway). Investigations are ongoing to detail all sourcing and distribution, and to identify the precise source of contamination in the materials.

From 22 September 2025 to 11 June 2026, Producer X supplied their non-organic pumpkin seed stock (ca 100 tons) largely to Supermarket A (no product was supplied outside this time period). Export was identified to Belgium, Switzerland and Luxembourg through the Rapid Alert System for Food and Feed (RASFF; no. 2026.7693). Switzerland and Belgium did not report any related human isolates (personal communication: Michelle Raess, 3 September 2026; EpiPulse, Belgium, 4 September 2026). Luxembourg reported a related human isolate on 3 September (Figure 1). The case was ill in late August and reported consumption of mixed nuts and raisins containing pumpkin seeds. Sampling of the leftover product confirmed contamination by the outbreak strain. The United Kingdom (UK) and Portugal also reported isolates in the outbreak cluster (Figure 1). The UK patient had travelled to France from mid-July to early August and reported symptom onset the week after return to the UK (no information available on pumpkin seed consumption; personal communication: Anne Hoban, 3 September 2026). The isolate from Portugal dates from May 2026; no information on a link with France or pumpkin seeds is available. Further distribution in France, as well as possible export to other countries in the European Union and European Economic Area through secondary distribution (below the wholesale level) is suspected and investigations continue. Recall messages and RASFF will be updated accordingly.

## Description of the ongoing outbreak 

Cases continue to be identified at the NRC-ESS for patients who fell ill before recall and withdrawal measures. In total, we have so far contacted 42 cases and conducted 25 interviews which confirm high rates of consumption of raw pumpkin seeds for 21 of 24 cases with available data, largely purchased at Supermarket chain A (19 of 21 cases). 

**Figure 2:**
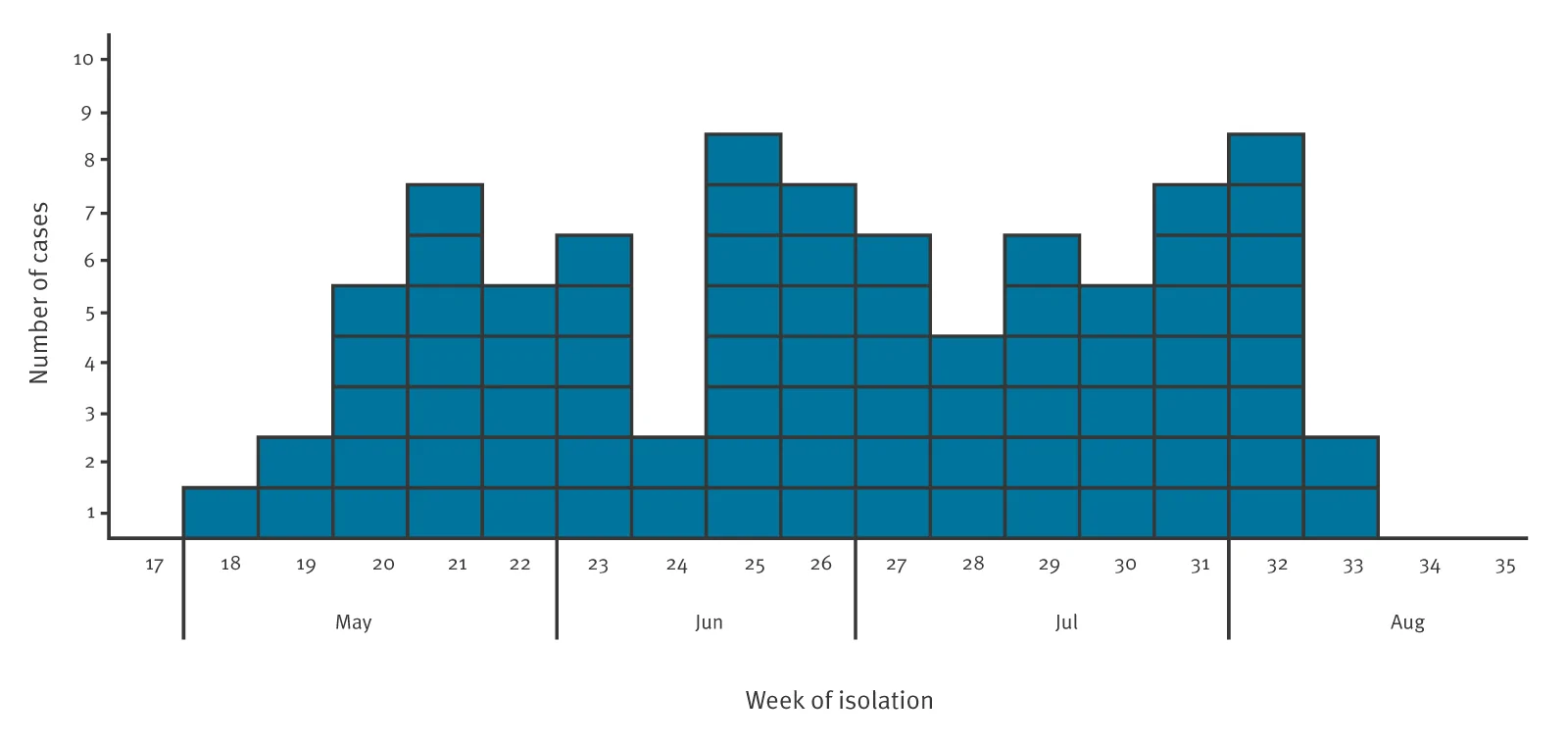
Outbreak curve of French isolates belonging the cluster of *Salmonella* Enteritidis HC2_710439, May–August 2026, France (n = 81; updated 9 September 2026)

**Figure 3:**
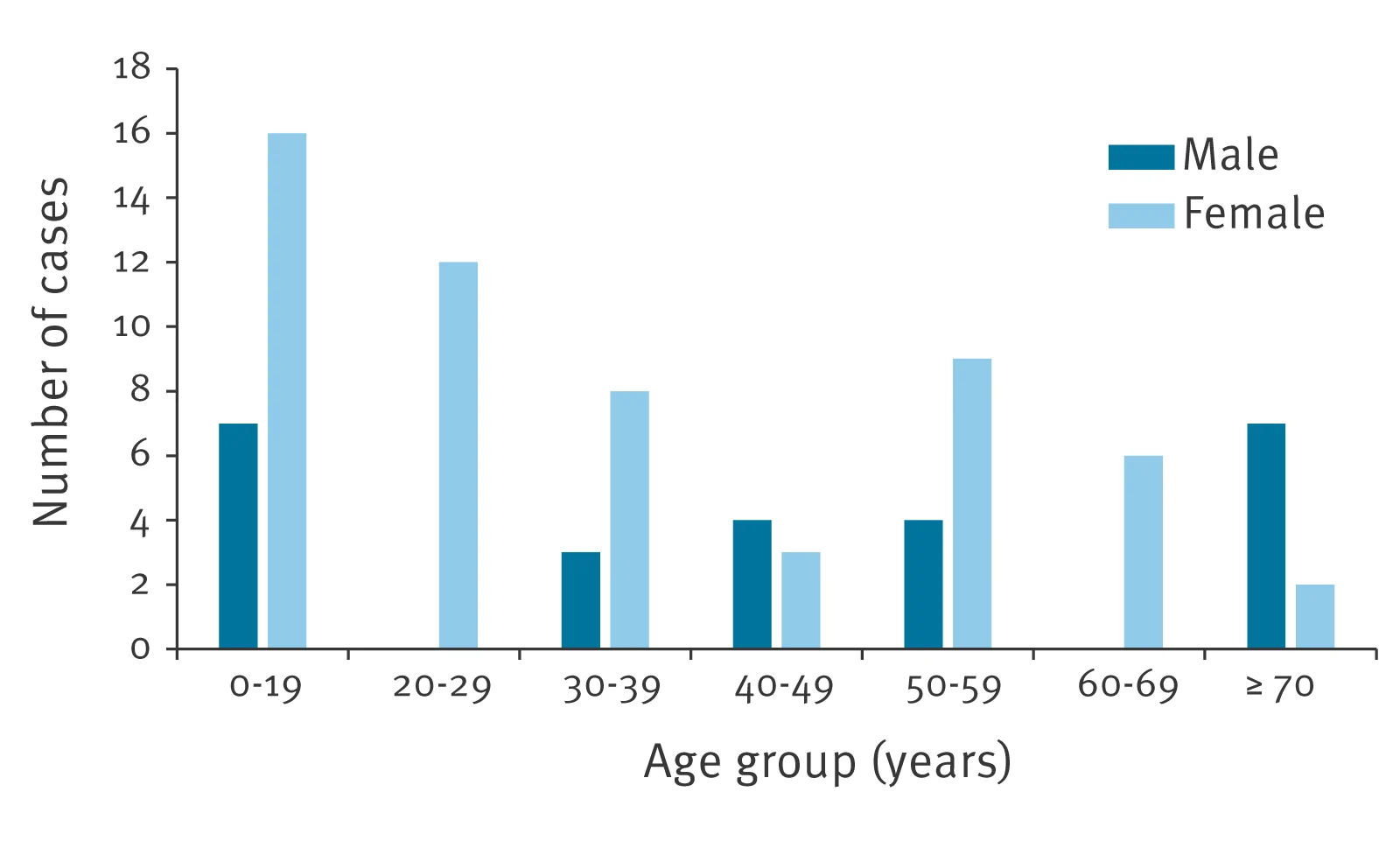
Age and sex distribution of cases belonging the cluster of *Salmonella* Enteritidis HC2_710439, May–August 2026, France (n = 81; updated 9 September 2026)

To date, 81 French isolates have been identified in this cluster, differing by 0–2 allelic differences (Figure 1). Isolation date ranges from 3 May 2026 to 12 August 2026 (Figure 2). Cases are predominately female (56 cases), and the median age has remained stable at 35 years (range: <1–83 years) (Figure 3). While one region remains disproportionally affected (28 cases), cases resided in 10 of 13 regions throughout mainland France. Hospitalisation data was available for 25 interviewed cases: eight were hospitalised and two consulted at emergency rooms.

Week of isolation corresponds to the date of *Salmonella* isolation at the primary laboratory.

## Discussion

We report a nationwide outbreak of *Salmonella* Enteritidis linked to consumption of pumpkin seeds. Epidemiological, microbiological and traceback investigations provided strong elements for confirming the source and implementing public health measures. We documented high rates of reported consumption of pumpkin seeds from Supermarket A and purchases on loyalty cards. Traceback confirmed the epidemiological link, identifying a single producer supplying Supermarket A. Finally, isolates from pumpkin seed samples belonged to the outbreak cluster, including an isolate from a European Union seed sample during the outbreak period and three samples from case homes. A further two samples from the French processing plant were positive for SE (sequencing underway).

Our investigations were limited by a relatively low response rate (25 of 42 cases contacted). However, the frequencies reported for purchases at supermarket A and for consumption of pumpkin seeds were sufficient to identify a hypothesis and initiate the necessary traceback investigations to identify the source. 

Pumpkin seeds have a long shelf life, are often consumed raw and are added as garnish or included in snack mixes, which complicates epidemiological and traceback investigations. Our trawling questionnaire was sufficient to identify a profile of cases consuming nuts and seeds, leading us to extend the questions regarding these food types. However, the response to the EpiPulse message reporting a related food isolate was key in reinforcing the initial hypotheses and identifying pumpkin seeds as the possible outbreak source. Loyalty card data were also instrumental in identifying pumpkin seed purchases. Expanding the data extraction period was key to capturing purchases of this shelf-stable vehicle.

While experimental studies have demonstrated prolonged survival of SE in pumpkin seeds [5,6], no outbreaks have been published in the literature. Several examples of pumpkin seed contamination have occurred recently, including recalls of pumpkin seeds for *Salmonella* contamination in Canada and the United States in 2025 [7-10], and the identification by Austria of a food isolate from import controls in June 2026 reported in EpiPulse. In both these cases, no human cases were reported in these countries. These elements together demonstrate the risk of *Salmonella* in pumpkin seeds, potentially from different countries of origin and distribution, and this French outbreak confirms the potential for large scale contamination in humans.

While European regulations impose upon manufacturers of ready-to-eat products such as pumpkin seeds the responsibility of respecting microbiological criteria, including for *Salmonella,* such novel food vehicles present a variety of challenges for public health and food safety [11]. 

## Conclusion

This nationwide outbreak identifies pumpkin seeds as a novel vehicle for *Salmonella* Enteritidis infection and demonstrates the value of combining whole genome sequencing, detailed case interviews, loyalty card data, food testing and international information sharing to identify uncommon, shelf-stable food vehicles. Pumpkin seeds and other ready-to-eat seeds typically consumed raw should therefore be considered in future *Salmonella* outbreak investigations, and strengthened controls throughout production and processing are needed to reduce contamination risks and prevent further outbreaks.

## Data Availability

All genomes and typing data were made publicly available in Enterobase. (enterobase.warwick.ac.uk). The isolate reference numbers are appended in the Supplement.

## Supplementary Data

34000 Supplement
